# Portuguese Older Persons’ Views about Living in a Nursing Home: Challenges to the Rehabilitation of the Image of LTC in Post-Pandemic Times

**DOI:** 10.3390/ijerph191710566

**Published:** 2022-08-24

**Authors:** Rute Lemos, Alexandra Lopes, Isabel Dias, Henrique Barros

**Affiliations:** 1 PhD Program in Sociology, Department of Sociology, Faculty of Arts and Humanities, University of Porto, 4150-564 Porto, Portugal; 2 Centre for English, Translation and Anglo-American Studies (CETAPS), 4150-564 Porto, Portugal; 3 Department of Sociology, Faculty of Arts and Humanities, University of Porto, 4150-564 Porto, Portugal; 4 Institute of Sociology, University of Porto, 4150-564 Porto, Portugal; 5 Epidemiology Research Unit, Institute for Public Health, University of Porto, 4050-600 Porto, Portugal; 6 Department of Public Health and Forensic Sciences, and Medical Education, Medical School, University of Porto, 4200-319 Porto, Portugal; 7 Laboratory for Integrative and Translational Research in Public Health, 4050-600 Porto, Portugal

**Keywords:** long-term care, nursing homes, older people’s views, violence and abuse, COVID-19

## Abstract

This paper addresses the broad topic of what older people think about nursing homes in Portugal. In the aftermath of the COVID-19 pandemic and considering the tragic events taking place in nursing homes, the challenge of reimagining the sector involves not only improving procedures and models of care, making sure they meet what citizens consider adequate, but also rehabilitating the image people have about nursing homes and rebuilding trust. Current and future decisions about how one meets LTC needs is influenced by the extent to which individuals see the alternatives as adequate. The paper presents evidence collected from a qualitative study run just before the COVID-19 pandemic began, with a sample of 45 community-dwelling individuals aged 60 plus, in Portugal. Opinions and views about nursing homes were collected and the results point to enduring negative aspects that are considered at odds with what constitutes adequate care. The paper discusses in length what those aspects are and concludes that future reforms of the nursing homes sector in Portugal need to consider what current and future users think and feel about that specific type of service. Debates in Portugal tend to be more focused on discussions about expanding the sector and less on aspects of quality of care. Views and opinions of interviewed participants, however, suggest that people may be more worried about quality of care.

## 1. Introduction

Older people across the world have been hit very hard by the COVID-19 pandemic and among these the very old (80+) living in nursing homes have been hit the hardest. Declarations by the Commissioner for Human Rights, in the Council of Europe, labeled as tragic what happened in European nursing homes because of the COVID-19 outbreak. Commissioner Mijatović’s further considered that the “(…) absolute priority right now must be to make sure that this experience is never again repeated over the course of the COVID-19 pandemic.” (https://www.coe.int/en/web/commissioner/-/lessons-to-be-drawn-from-the-ravages-of-the-COVID-19-pandemic-in-long-term-care-facilities (last assessed on 7 July 2022). The pandemic seems to have brought a heightened sense of mistrust in how some Long-Term Care (LTC) services operate and views about how safe residents are when admitted in such facilities are grim [1]. It has also added pressure on debates about the future of LTC. In the aftermath of the COVID-19 crisis, nursing homes have been faced with the challenge of improving their procedures to deal with public health emergencies and disasters, but they are also faced with the challenge of rehabilitating their image to regain the trust of the population [2]. In Portugal the experiences of COVID-19 in nursing homes were not very different from what was experienced elsewhere, with a general consensus that events unfolded with very negative consequences for residents [3,4].

In this paper we address the broad topic of what older people think about nursing homes in Portugal. We address more specifically how older people perceive nursing homes as places of high risk for violence and abuse and why that is the case. We argue that COVID-19-related events have provided additional evidence for what many older people already thought about nursing homes in Portugal before the pandemic [5]. The views about nursing homes in pre-pandemic times were already dominated by images that portrayed them as places of violence, abuse and violations of fundamental rights and freedoms [6]. However, even the most negative views about nursing homes did not consider the possibility of abandonment and neglect that was experienced in some locations during the COVID-19 crisis but rather focused on broad notions of dignity, freedom of choice and emotional well-being [7]. In that sense, the COVID-19-related events have potentially made worse a view that was already negative.

In this paper we present evidence collected before the pandemic from the discourses of interviewed Portuguese older persons and we discuss the aspects involved in their views of nursing homes as places of violence, abuse and substandard care. We further discuss the implications of views about nursing homes for policy development related to formal LTC reforms and improvement. The central claim of the paper is that the need to rehabilitate the image of nursing homes in the aftermath of the COVID-19 pandemic must involve a consideration of the views citizens have about these services. Furthermore, the authors claim that (re)building trust in nursing homes is not just about addressing the specific issues brought about by COVID-19 but also about addressing some long-lasting aspects of how citizens believe nursing homes operate.

## 2. Literature Review

The literature on the views people have about nursing homes is dominated by studies that take a specific angle of analysis both in terms of whose views are considered and which topics are addressed. As far as the populations of reference are concerned, most studies have focused on the views of those already living in nursing homes, which means that views are most likely shaped by the individual experiences as residents of such facilities [8,9]. Several studies have also considered the views of relatives of residents [10] while others have focused on the views of professionals working in nursing homes [11,12]. Topics addressed tend to revolve around the theme of satisfaction with care provision, relationships between residents, residents and staff and/or relatives. There is a general consensus about some mismatches between what residents want and what residents receive [13] as well as about the challenging nature of nursing homes as workplaces [14,15].

There are not many studies addressing the views of the general population or of specific age groups about nursing homes. The ones that are published focus on how individuals appraise and value different LTC arrangements in view of discussing preferences and grounding decisions about what constitutes adequate LTC provision and financing. The literature review carried out by König and his team on this topic highlights the consistency in findings across studies suggesting that most people prefer to receive LTC in their own home when care needs are moderate but switch to residential care when care needs are extensive [16].

Understanding what individuals think about different LTC arrangements is a central tenet of a person-centered approach to LTC planning and organization. Stakeholders across the ecosystem of LTC seem to speak with one voice when claiming the need to secure LTC systems evolve in manners that safeguard the personal and social identity of users, their independence and autonomy, guaranteeing that individuals stay in control of their lives and live with dignity even when they become severely dependent [17]. Some studies have suggested that LTC preferences are a function of the perceived ability of a specific LTC arrangement to satisfy peoples’ basic physiological and mental/social needs [16]. Mapping the views individuals have about different LTC services then becomes very relevant as it can offer valuable information to understand expectations individuals are likely to carry when their time comes to secure care that tackles their needs.

One topic that can be found in the literature is that of nursing homes and institutionalization being often viewed at odds with the safeguard of fundamental rights and freedoms, largely due to the model of care provision in place, highly standardized along the lines of block-treatment and with little to no room for consideration of individual identities, preferences, and expectations [18]. Studies on cultures of work in institutional care facilities have highlighted how thin the barrier is between poor quality and violence in what concerns the relationship between residents and their caregivers [19]. Some research has paid particular attention to the identification of factors that increase the risk of violence in nursing homes, with results pointing to dementia as one of the most critical ones [20,21]. Studies on service provision quality have signaled the occurrence of situations of neglect in nursing homes, often associated with understaffing [22] and lack of training of staff [23]. To all these we can then add the myriad of risk factors directly stemming from the socioeconomic and relational vulnerability of residents, from their health conditions and heavy needs for care [24,25]. Directives to improve the quality-of-care provision often include references to the need to implement mechanisms of prevention of violence and abuse, which in a way means that the risk is officially recognized.

## 3. Materials and Methods

### 3.1. Research and Design Methods

This paper reports on the partial results obtained from a qualitative study carried out as part of a PhD thesis in sociology, which in turn was carried out integrated in a broader project named HARMED. HARMED was a large research project carried out in Portugal that addressed the topic of violence and abuse of older people looking at both the socioeconomic determinants of the phenomenon, its prevalence and its consequences. HARMED involved a mix-methods research design, combining a survey-based research approach with a collection of health markers by means of physical examinations, including blood collection and with a subsequent qualitative study. It was carried out between 2017 and 2020 by a multidisciplinary team of researchers that included social scientists, policy analysts and public-health researchers. Data reported in this paper proceeded from the qualitative study carried out within the HARMED project which involved the collection of discourses with in-depth semi-structured interviews to 45 community-dwelling persons aged 60 years old or older, with intact cognitive functioning. Material collected in the interviews offered the empirical corpus that would be used for the PhD thesis.

The goal of the qualitative study carried out within the HARMED project was to explore the narratives and discourses of older people to understand what individuals define themselves as violence and abuse, how they explain the occurrence of such phenomenon and who they consider are the perpetrators of violence and abuse as well as the responsible agents for safeguarding older people. One of the research questions that researchers from the HARMED team were interested in exploring was to what extent the definitions and views of older people mirrored the official definitions, policies and mechanisms in place in the country to address the violence and abuse of older people.

The collection of views, experiences and personal narratives was prepared along the lines of an exploratory approach, aiming at unravelling the spontaneous discourses of interviewees and hence the views they would more easily express without guidance from the interviewer. The interview check-list was therefore only semi-structured, and questions were asked in a very open way. Interviewers were instructed to pursue any theme the interviewee raised and explore his/her views on the how’s, why’s, who’s and where’s of all narratives about violence and abuse of older people.

The topic of nursing homes was not in the original design of the qualitative study, or as a matter of fact, in the research design of HARMED. In reality, the research framework was much more focused on family dynamics and violence taking place therein. The topic of nursing homes showed up spontaneously during the course of the research and it was its quantitative expression, mentioned by more than half of the participants, that turned the eyes of the researchers to the theme, which would become a research topic in its own right.

Interviews of the qualitative study were completed before the COVID-19 pandemic, during 2018 and between the months of April and May. 

### 3.2. Sample and Procedures

The 45 interviewees that were included in the qualitative study were selected from the sample of individuals that participated in the first phase of the HARMED project. That sample included 677 persons aged 60 plus living in the community in the region of Porto, in Portugal which in turn had been selected from the on-going cohort of residents in the Porto region that have been followed since 1999 in the EPI-Porto study [26]. Criteria for selection of interviewees included demographic characteristics (gender and age) and exposure to violence and abuse. In the first phase of the HARMED project a survey was used and information about the status of participants concerning exposure to violence and abuse was collected. The final sample of interviewees therefore included individuals of different ages and genders as well as individuals that had already had personal experiences of violence and abuse, of different types, and individuals who had never had any such experience.

Interviews took place in the research office of the team located in the facilities of the main hospital in the North region of Portugal. The partnership with the Institute of Public Health facilitated the use of hospital facilities that participants already knew from previous cohort evaluations. Interviews were audio-recorded and later transcribed. The duration of interviews ranged from 27 min to 3 and half hours. 

The HARMED project, including the qualitative study, was approved by the joint Ethics Committee of São João University Hospital Center and University of Porto Medical School. Written and signed informed consent prior to the interview was secured from all participants with an explicit declaration of authorization to audio-record the interview.

Senior researchers from the HARMED team were in charge of completing the interviews as the interviewing protocol, due to the topics covered, required experienced interviewers and a very detailed knowledge about the research topics and about the findings from previous phases of the HARMED study.

### 3.3. Data Analysis

Content analysis of the transcribed interviews was carried out with the help of NVivo Version 12 and version R 1.6. The strategy implemented for the analysis resorted to a protocol of double coding to control for coder’s bias and to improve the reliability of classifications. The text was first coded following the structure of the interview check-list and the topics raised by interviewers. The work then proceeded with the coded extracts that were subsequently analyzed for content in view of identifying the emerging themes and the associations between themes and ideas.

## 4. Results 

In this section we report the results from the qualitative study that relate to the opinions, expectations and images the participants shared about nursing homes when asked to talk about the meanings of being old in today’s society and in particular about the risks of and the occurrence of violence and abuse against older people.

As a general note, it must be highlighted that participants were not specifically asked to talk about nursing homes. They were asked about what their experiences, knowledge and opinions about violence and abuse against older people were. Nursing homes emerged spontaneously in the discourses of participants and once the topic was raised interviewers would pursue it probing interviewees to expand and explain their views. Among the 45 interviewees, 23 (51%) spontaneously raised the topic of nursing homes when asked about violence, neglect and abuse against older people. This was the second topic most frequently raised by participants along the interviews. Although the totality of participants identified the family as the main locus of violence and abuse against older people, with 40 out of 45 declaring that the main abusers of older people are their adult children, nursing homes came second before other topics such as violence by strangers (19 out of 45 talked about criminal assaults), violence by the state and society in general (14 out if 45 have talked about discrimination of older people and how badly people in general treat older people) and violence by intimate partners/spouses (5 out of 45). In this paper we present the results of the analysis carried out on the parts of the interviews that involved views about nursing homes.

The presentation of the results is organized according to the categories of analysis that emerged from the content analysis protocol. Those categories included: (i) the representation of admission to nursing homes as a violent experience; (ii) the poor quality of care in nursing homes as a form of violence and neglect; (iii) the understanding of nursing homes as places where fundamental rights and freedoms are violated; (iv) the view of nursing homes as places of reproduction of social inequalities that aggravate the risk of abuse, violence, and neglect.

### 4.1. General Characterization of the Interviewed Participants

The 45 participants in the qualitative study included 20 males and 25 females, with ages ranging from 60 to 87 years old and living in the community. Around half of the participants had less than secondary level education. Most participants were married and with a living partner (73%) while for others the partner was deceased (20%). The remainder were either single or divorced.

Selection of participants for the qualitative study was completed from the pool of individuals that had previously participated in the HARMED survey [27]. From the survey database it was possible to select individuals that had declared having been victims of violence and abuse in the 12 months before the survey as well as individuals who had declared not having had such experience. Among the 45 participants in the qualitative study, 27 (60%) had been victims of violence and abuse. The other 18 participants had not. Table 1 displays the frequency of different types of violence among the participants who have declared such experience.

Of the 23 participants that spontaneously raised the topic of nursing homes while sharing their views about violence against older people, 15 (55.6%) stated that they had been victims of violence and abuse in the last 12 months. This figure is particularly relevant in the context of this paper since it dismisses the hypothesis of negative views about nursing homes being a function of one’s own experience as victim. The experience of victimization in the total sample of participants was actually higher than that recorded among the ones raising the topic of nursing homes.

Additionally, the distribution of those mentioning nursing homes in the course of the interviews was also controlled for gender, age and marital status. No significant association was identified. There were some differences associated with schooling levels and income, but the non-linearity of the association suggests the effect may be spurious. Although this was a qualitative study primarily concerned with unravelling aspects of what people think, and not so much with estimating prevalence of any given view, the fact that there was no evidence of results being skewed by demographics, socio-economic conditions or personal experiences of violence speaks to the validity of the results discussed in the paper as signaling common views and opinions.

In the following sub-sections, the paper summarizes the main aspects that were present in participants’ discourses. Examples of statements included in the interviews are included in the results.

### 4.2. Admission to Nursing Homes as a Violent Experience

Association of nursing homes to violence showed first in discourses tied to descriptions of the family as an agent of violence. Participants saw the integration of older people in nursing homes as a show of abandonment and emotional violence from the family.

*«Very often they [adult children] leave the elderly there [nursing homes] and don’t even go visit.»*

*(Interviewee 22)*

*«In the past you were born at home and you would die at home. Nobody want to leave their house. (…) Anyways, we have our place, our space, our things, our world and leave here to be locked in some place where everything is missing and where the children never go or go very seldom.»*

*(Interviewee 33)*

Norms on family solidarity and filial obligations were very explicitly stated by some interviewees, with the violation of those norms being equated with acts of violence and neglect.

*«The place of an older person is next to their children because a parent gave everything for a child and the adult child has the obligation to look after the parent.»*

*(Interviewee 19)*

*«It hurts me to see an old lady, a neighbour of mine, very nice lady, her only son is a medical doctor and he placed his mom in a nursing home. You can’t say it’s because he doesn’t have economic resources. It’s because he doesn’t want to put up with her.»*

*(Interviewee 33)*

*«I know people here in Porto that their adult children, when those people reach a certain age, they will not want to put up with them and will take them and put them in nursing homes. And those nursing homes are what we all have been seeing, right?»*

*(Interviewee 31)*

Interviewed participants expressed their negative views about how the process of admission into nursing homes was managed by the families. They considered that for many older people it is a forced admission that does not match their preferences but is rather imposed by the family, often with very negative consequences for their well-being.

*«There was this lady that lived in her house and then the son came and put her in a nursing home. Some days later she died of sadness. She didn’t want to go.»*

*(Interviewee 19)*

Faced with the prospect of ever needing care and being forced to go into a nursing home, participants expressed their anxiety in association with their negative views of those institutions as places of violence.

*«In nursing homes they treat older people very badly, because I have been to many nursing homes and I come back feeling sick. All nursing homes, all, no matter which one. That is why I feel very sad and I ask God that I don’t want to go into a nursing home… I will only go if they come and take me, I prefer to die in my bed.»*

*(Interviewee 9)*

### 4.3. Poor Quality of Care in Nursing Homes as a Form of Violence and Neglect

Nursing homes were described as places of violence and abuse in their own place as well. Two types of violence were associated with nursing homes as a result of how care delivery takes place: physical violence and psychological violence. Those working in nursing homes were identified by participants as the agents of violence, although not necessarily due to personal characteristics or traits of personality but mostly due to the working context where they were integrated and delivering the care.

Views about the quality of life of residents in nursing homes were very negative, with participants voicing concerns about the substandard care older people receive and about the consequences that has for their well-being.

*«There were certain signs that she was not well treated. She has these earrings, small golden earrings that I had offered her, like a little ring, and one day when bathing her the earring broke and so on when they were drying her. I thought that they had a very rough way, very stupid let’s say, to treat her. »*

*(Interviewee 2)*

*«It’s enough to see the way they lift the bed linen and how they turn that old person to the side to wash him and clean. And they start with ‘you, common, turn around’»*

*(Interviewee 36)*

*«The experience I have of nursing homes that are in the news is that they are very bad, very bad, where you see physical and psychological violence against older people which makes my stomach go around. I see them strapped to the beds, with pressure ulcers, with no medical assistance.»*

*(Interviewee 20)*

Participants saw these events taking place mostly due to the poor quality of the staff that worked in nursing homes, who were considered as lacking appropriate training, and due to the working conditions offered in these institutions. Some of the interviewed participants went further, acknowledging that looking after an old person presents itself with challenges that make the job unattractive and without proper training this is likely to increase the risk of the caregiver not doing a good job and ending up being physically violent or abusive to the residents as they compromise the physical well-being of residents. There were concerns as well about the scope of services that are available, namely about the lack of regular medical assistance.

*«Many of the staff do not get paid enough or they don’t have appropriate training.»*

*(Interviewee 26)*

*«It’s very few the nursing homes that are well equipped and organized with staff capable of giving life to those persons and secure good quality of life till the end.»*

*(Interviewee 33)*

*«Now, does a nursing home have a medical doctor? Is there one that goes there to see the residents? Do they have any nurse? It’s the lady that takes care of the cleaning that cleans pressure ulcers and changes bandages? »*

*(Interviewee 33)*

*«In nursing homes we are looked after by people who have no relationship with us. If they are not given proper training, what do I get if I go to a nursing home and the staff doesn’t feel love or affection to me, it’s not like I do with my mother, that I love her. »*

*(Interviewee 22)*

*«The nursing home facilities may be great, we look at the nursing home and it looks great, but the staff is not trained to deal with older people. »*

*(Interviewee 36)*

Substandard care was also mentioned in association to experiences the participants label as psychological violence. Topics that were mentioned by interviewees included the way the staff talk to residents, resorting to shouting and rude expressions, but also the general effects of a block-treatment model that fosters depersonalization and an approach to life that participants see as meaningless and sad.

*«Food must be the same for everybody, that place is almost like military quarters. You get up, you go eat, you sit in the armchair, you see TV or you play cards, or do lace. At lunch time they eat what they are served—you want it, you want; you don’t, you don’t eat. »*

*(Interviewee 22)*

*«I think that in nursing homes there is a lack of love, lack of tenderness, lack of understanding.»*

*(Interviewee 33)*

*«[about a nursing home the interviewee has visited] Residents had to get out of bed early in the morning, they couldn’t stay in bed because the staff want to bathe them, they want to clean the bedrooms. I agree that the resident gets up when they are able to do it, but not that early, but they are forced. »*

*(Interviewee 9)*

*«They don’t have any activity, and they stay there all day sitting in a sofa, in a chair, sleeping, looking at the floor.»*

*(Interviewee 7)*

*«I find it hard looking at nursing homes and seeing them [the residents] there waiting for the time, the day, the hour [of death], pushed, cornered… I looked at all that, all those people, I mean, abandoned, alone, with no activities, with nobody to motivate them, just waiting for the time to arrive.»*

*(Interviewee 33)*

### 4.4. Nursing Homes as Places of the Violation of Fundamental Rights and Freedoms

A recurrent topic in participants’ narratives about violence against older people associated nursing homes with the violation of fundamental rights and freedoms, seen as a form of violence. Dimensions of rights more frequently addressed included freedom of choice and autonomy. Nursing homes were viewed as places where the organizational model does not safeguard the autonomy and freedom of choice of residents, and sees all aspects of their daily lives decided by others with no room to accommodate their preferences and expectations. Managers of nursing homes were pinpointed as the main agents of aggression here. They were described as being primarily focused on the commercial aspects of running a nursing home, prioritizing profit over the well-being of residents. Lack of transparency in how nursing homes are managed, coupled with the absence of an independent national quality control system were also mentioned as aspects concurring to exposure of residents in nursing homes to risks of violence and abuse.

*«The news we ear is that if a person goes there [nursing home] and says yes to everything, all is good. If you are a bit cheekier and you feel this or that is not right, what do they do to you? They stuff you will pills for you to spend time sleeping and soon you leave this world.»*

*(Interviewee 22)*

*«[nursing homes] have commercial goals and they are there to see how fast people die, the faster the better because another resident will come.»*

*(Interviewee 33)*

*«If they [older people] have money, what do they do? First thing is to check what assets they have and pass everything to the nursing home. I am talking about a nursing home I know very well [participant identifies the nursing home by its name but omitted from the paper due to data protection legislation]. I had a friend who was well-off and he went to that nursing home. He didn’t want to go, he wanted to stay with his children, but ok they didn’t want to be bothered. He went to that nursing home and the first thing the management did was put all his assets as property of the nursing home. And my friend was a person with possessions. »*

*(Interviewee 18)*

*«We had to warn the day before that we were going to visit my mom and she would show up with her hair dyed, and she only complained that she was being starved.»*

*(Interviewee 29)*

### 4.5. Nursing Homes as Places of Higher Risk for the Most Vulnerable

One last theme that emerged in participants’ discourses about nursing homes was the concern with the most vulnerable older persons, perceived as being at particularly high risk of being victims of violence and neglect once admitted into nursing homes. Vulnerabilities were described from different angles. Some participants linked this topic to the focus on profit leading to a lower quality of care provided to residents with lower income. In that sense, nursing homes were described as reproducing the classic socioeconomic inequalities, aggravating them since their substandard care put at risk the health of residents affected (five interviews). Some participants voiced concerns about the situation of residents with more severe health conditions, namely those with cognitive impairment and/or those who were dependent on assistance for basic activities of daily living. Residents with these conditions were described as lacking the ability to voice their discontent or resist what was imposed on them, even if against their will (two interviews). A few interviewees highlighted the even higher risk of those living in nursing homes that operate illegally and without licenses.

*«Sometimes, in nursing homes, my friend told me that it’s not really aggressions nor very concrete cases, but it’s a feeling that there is an exploitation, not only economic, but also emotional from those in charge of managing the nursing home. Especially with those who have no autonomy. Because those who have autonomy, like my friend, they go out, they go for a walk, to the coffee shop, and then they return. But he tells me that other residents there, the majority, they just sit and look at the TV all day.»*

*(Interviewee 6)*

*«From what I have seen, I think people who have certain illnesses, Alzheimer and so on, those are not well treated. If in those place they treat people well it will be those that know how to say things and complain to their relatives that pay money and don’t want them badly treated,»*

*(Interviewee 2)*

*«There are illegal nursing homes and these smart ladies of the money… and then there are workers that come out and denounce that the residents are malnourished, not having proper hygiene.»*

*(Interviewee 38)*

*«Nursing homes are overloaded, people have to wait a long time for a place from the Social Security, and sometimes when something comes up it is very bad.»*

*(Interviewee 39)*

«But there are also good nursing homes where you pay a lot of money. And a person with a pension of 370 euros per month, how can that person afford a good nursing home? They have to go to a sh***ty nursing home.»

*(Interviewee 22)*

## 5. Discussion

In this section we discuss the implications of what was presented as being the opinions, expectations and images the participants had about nursing homes and in particular of the rationale involved in the association of this specific LTC setting to violence and abuse. The section is split into three separate subsections that address the two main domains of implications authors want to emphasize and the limitations of the study. Firstly, we highlight the aspects of quality and adequacy of care (or lack hereof) in nursing homes that populate older people’s views and opinions, and we link those to ongoing discussions about the future of LTC. Secondly, we discuss the implications of findings for discussions on rehabilitation of the image of nursing homes in post-pandemic times. On this we highlight in particular the importance of linking the discussion to the agenda of fundamental rights and freedoms going beyond the strict limits of public-health debates. Finally, we set the findings into context acknowledging the limitations of the research design.

### 5.1. Quality and Adequacy of Care in Nursing Homes

That Portuguese older people, in general surveys, display a low inclination towards meeting their care needs by resorting to admission into nursing homes is not a novelty. Research on LTC preferences has systematically shown that their preferred LTC arrangement still goes to family-based solutions [28]. Most discussions about this stated preference tend to emphasize the family-oriented social model in place in Portugal, with resilient social norms that sustain notions of filial obligation and family network closeness [29]. More recent discussions have shown that this normative framework has been changing, which translates into feelings of disappointment on the side of older people confronted with families that struggle (often with no success) to fulfil traditional expectations about family solidarity and intergenerational relationships [30]. Although there are anecdotal reports about nursing homes being bad places where people are not well attended and where residents feel unhappy, there is not any research linking aversion to nursing homes among Portuguese older people to notions about how they operate and about how life unfolds for those living there.

The findings of the qualitative study that were reported in this paper shed some light on this topic and raise some issues that may have potential as explanatory elements of the continuous resistance of older people, in Portugal, towards LTC arrangements that involve institutional care. Furthermore, the findings presented in the paper offer new evidence about the importance of addressing quality of care in nursing homes in policy discussions about LTC in Portugal and show that potential users of LTC are not just worried about quantity of care available, as some discussions on the need to expand the sector seem to suggest but are also very worried about the quality of care that is offered.

The images voiced by the participants in the qualitative study continue to align with the literature that classifies the Portuguese welfare state as part of the family-based model, since admission into a nursing home is still described as something that happens as a failure of families and moreover as an expression of erosion of family ties. This is a view that comes hand in hand with a negative opinion about how society has evolved. Nursing homes, in that sense, are still viewed as the place where those who have nobody truly caring for them go. Abandonment is therefore the dominant feeling associated with admission into a nursing home. Although not new, the resilience of the image of nursing homes as asylums is likely to continue affecting older persons’ decisions when the time comes to choose how to tackle needs of care and assistance. It is also likely to increase the pressure on the demand side for arrangements that prolong staying in the community and in one’s home.

The findings, however, go beyond the association of going to a nursing home to being abandoned by the family. This, one could argue, is an image very much centered on what a person feels the future may hold for himself/herself and can represent some feeling of personal disappointment. The findings suggest that people have a very negative view about the quality of care found in nursing homes and the aspects they raise are very much in line with some ongoing debates at the policy-making levels on the challenges ahead for LTC, not only in Portugal, but elsewhere [31].

Firstly, people are increasingly aware of how models of care provision and cultures of care affect the quality of life of those living in a nursing home. Even if participants do not use the scientific jargon, their narratives address issues of standardized care, block-treatment and depersonalization. These are issues that worried the participants in the study and that were labelled as conducive to practices that they saw as violent, if not always physically violent, certainly psychologically and emotionally violent. Reports of known cases, of relatives and acquaintances, suggest that these views are grounded in real experiences and not merely the reproduction of what is heard in the news.

Secondly, people are increasingly aware of the importance of integrating social care and healthcare. Portuguese stakeholders with responsibilities in the process of policy-making in the LTC sector have long advised that nursing homes should be considered as an arrangement that should be used when the person has levels of dependency that make it almost impossible to remain at home. Indeed, what we have seen over the last two decades is the consolidation of a specific profile of resident that is typically very old, over 80 years of age, with cognitive impairment and/or severe dependency in activities of daily living and personal care [32]. These are individuals that tend to accumulate several health conditions often requiring healthcare. Yet, in the Portuguese social care system, nursing homes are still not required to include healthcare staff in their teams, something that only happens at the discretion of the promoter. What the images voiced by the participants in our study show is a heightened sense of how inadequate this is and how the lack of full integration of social care and healthcare in nursing homes puts at risk the well-being and even the safety of vulnerable residents.

Thirdly, discourses from the participants in the study reveal that people are aware of the close association between quality of care and labor dynamics in the sector. If it is true that the negative image the interviewees had about the labor force working in nursing homes reflects a general opinion about how society treats older people, it was also possible to verify that people considered that the root problem involves matters of selection of workers, training opportunities, salaries and working conditions in general. Although this was not presented as an excuse for the occurrence of instances of violence and abuse, participants were aware that the way forward to improve quality of care in nursing homes will need to involve significant changes in the labor force involved in the sector. This is one of the most pressing topics one finds in reports from the OECD or from the EU when the discussion is about the future challenges of LTC: shortages of labor force; lack of training; low salaries; poor working conditions [33]. Our findings suggest the population may be very much aware of these things and is likely to pressure and be sensitive to these issues.

Lastly, the findings presented in the paper call for a systemic approach to the problems voiced by participants. Situations of violence, abuse and neglect are seen as facilitated by a system of care provision that is very weak in terms of the implementation of mechanisms of quality control and monitoring, an issue that has already been addressed by some national research [34]. Older people living in nursing homes are represented as vulnerable and therefore requiring protection from the state. Developments in social care systems across Europe have shown the potential of implementing effective, independent and transparent quality control mechanisms in pursuit of better quality of care [35]. Participants, themselves the potential future clients of nursing homes, were very much in tune with this approach and are likely to pressure towards developments in that direction.

Overall, what some would consider the surprising relevance of nursing homes in discourses of older people about violence and abuse, shows that there is very low tolerance to substandard care to the point of that being equated to violence and abuse. What this shows is the absolute importance of including in official discussions about formal care, in Portugal, what people think about existing services and what people consider (in)adequate in terms of service provision. The belief that older persons are more concerned about the long waiting lists and the low number of places available in nursing homes may be inaccurate. People are also very much aware of quality issues, and they are likely to be less and less inclined to take just what is offered. Pressure towards better services is therefore likely to climb in the near future and policy makers need to be aware of that.

### 5.2. Reabilitating the Image of Nursing Homes and the Agenda of Human Rights

Following the discussion put forward in the previous paragraphs and turning now to current times and to ongoing debates about the future of nursing homes post-COVID 19, what the findings from our study reveal is that efforts to rehabilitate the image of nursing homes may be more demanding than what some public health discourses suggest. Indeed, it may be insufficient to address the problem by means of the revision of protocols in place to deal with health emergencies. It may be necessary to revise the entire model of service provision and look at it from the perspective of human rights.

The first thing to remember is that the discourses were collected before the pandemic. The identification of nursing homes in association with aspects of life that participants consider forms of violence was very high and explicit. Considering the events that took place in nursing homes during the COVID-19 crisis, often accompanied by very graphic reports by the media, the negative images that were collected in the study would most likely be even worse and voiced by more people had the study been conducted after the pandemic. This is relevant for two reasons. On the one hand, it means that negative views about nursing homes already existed and were not a consequence of COVID-19-related events. Stakeholders with responsibilities in the sector often praise COVID-19 for making more explicit and visible to the general public what was already taking place before the outbreak [36]. Participants in the study seem to be a proof of the veracity of such claim. On the other hand, it means that COVID-19 has worsened what was already a very grim picture, which is likely to influence people’s decisions about LTC arrangements in the future and raise their awareness levels about issues of quality of care.

However, what the findings from the study also show is that there is a growing awareness of aspects of service provision that directly stem from the framework of fundamental rights and freedoms. The times when Portugal was a country where the population had not developed a culture of citizenship that put pressure on compliance with rights may be a thing of the past. Participants in the study seemed very much aware of the fact that autonomy, freedom of choice, respect, dignity, person-centered care, freedom of circulation and inclusion in society are fundamental rights that cannot be violated. Classifying models of care provision in place as forms of violence shows that precisely. Moreover, what this means is that the way forward for nursing homes in Portugal is going to be non-satisfactory if post-pandemic changes are only addressed from the angle of health prevention and safety protocols. A broader debate about the entire model of operations in these places is most likely needed and there are benefits in mapping how people currently view the sector to elucidate what and how changes are to be implemented.

### 5.3. Limitations and Future Research Directions

The research findings discussed in this paper have been obtained within a larger project that focused on the topic of violence and abuse of older persons in Portugal. In that sense, and although the theme of nursing homes emerged spontaneously among interviewees, the fact that they were being questioned about violence and abuse makes it more likely that the topic of nursing homes, when addressed, would be addressed in relation to violence and abuse. In other words, the evidence put forward in the paper was obtained in a research setting that did not create any opportunity for positive discourses on nursing homes to be detected. As such, one cannot infer from the current study that there are no positive views about nursing homes. If anything, the identification of the research topic as relevant points to the need for future studies examining our conclusions in a research setting that starts from a broader research question about nursing homes and not from a research question about violence.

Similarly, it is likely that choices in terms of sampling influenced the diversity of opinions that were collected about nursing homes. The sample of interviewees only included community-living older persons that had no personal direct experience of living in a nursing home. Looking at the Portuguese LTC landscape, the latest figures for places available in nursing homes allow us to estimate that 4.2% of the population aged 65 plus are living in a nursing home (Source: authors’ estimates based on resident population 65 plus and on official administrative data for the number of places in nursing homes licensed by the Social Security services, obtained from Carta Social). A potentially interesting line of research for future studies would be comparing the views of those living in institutional care settings with the views of those living in the community.

## 6. Conclusions

The COVID-19 pandemic has disproportionally affected nursing homes and although the individual health condition of residents is generally considered as a risk factor, debates about how nursing homes operate and about the quality of care delivered have increased amidst the tragic outcomes that were recorded around the globe in morbidity and mortality associated with COVID-19. In post-pandemic times, rehabilitating the image and the trust of the population in nursing homes is a challenge that must be tackled in a comprehensive manner considering not only the revision of healthcare protocols and response procedures to health emergencies, but also considering the broader aspects of what constitutes adequate care. In fact, while knowledge related to caring for residents during COVID-19 accumulated and fed decisions about what strategies should be pursued, meeting the expectations and demands of current and future residents requires a consideration of aspects of quality of care that were already seen as substandard before the COVID-19 outbreak. Aspects requiring responses will be better met with targeted strategies that also consider the views people have about nursing homes. Strategies to rehabilitate the public image of nursing homes are needed and in order to arrange for those it is important to start from what people know and think about nursing homes.

In that respect two main ideas have stemmed from the discussion put forward in this article based on the evidence collected from a sample of Portuguese older persons.

On the one hand, negative views existed already before the COVID-19 crisis and aspects of substandard care offered by these services were outlined very clearly even before people were exposed to the media coverage the sector had during the COVID-19. This points to views and feelings about nursing homes being more consolidated in people’s minds than what a mere reaction to a contextual event would suggest, which in turns sheds light on the size of the challenge ahead if we want to improve these views and feelings.

On the other hand, it is safe to assume that COVID-19-related events may have worsened the views people had about nursing homes. This is likely to play a role in older persons and their families’ decisions about how to tackle needs for care and therefore must be considered when planning future developments in the LTC sector.

## Figures and Tables

**Table 1:** Types of violence experienced by participants victims of violence (n = 27).

Type of Violence	n	%
Physical	7	25.9
Psychological	21	77.8
Financial	9	33.3
Sexual	3	11.1

## Data Availability

Not applicable. All data are presented in the article.
